# Effect of Low-Molecular-Weight Hyaluronate-Based Nanoparticles on the In Vitro Expression of Cartilage Markers

**DOI:** 10.3390/ijms252312486

**Published:** 2024-11-21

**Authors:** Annalisa Bianchera, Paolo Borghetti, Francesca Ravanetti, Laura Bertocchi, Elena De Angelis, Ruggero Bettini

**Affiliations:** 1 Food and Drug Department, University of Parma, Parco Area Delle Scienze 27/a, 43124 Parma, Italy; laura.bertocchi@unipr.it (L.B.); ruggero.bettini@unipr.it (R.B.); 2 Department of Veterinary Science, University of Parma, 43124 Parma, Italy; paolo.borghetti@unipr.it (P.B.); francesca.ravanetti@unipr.it (F.R.); elena.deangelis@unipr.it (E.D.A.)

**Keywords:** hyaluronic acid, low molecular weight, cartilage differentiation, nanoparticles

## Abstract

Hyaluronic acid (HA) is a key component of synovial fluid as it plays a crucial role in joint physiology. Its biological activity is influenced by molecular weight, local concentration, and persistence in joints. High-molecular-weight HA has a consolidated history of clinical use, whereas little is known about the metabolic effect of low-molecular-weight hyaluronate on cartilage differentiation. This study explores the potential of HA-based nanoparticles (NPs) on chondrocytes differentiation in vitro. Starting from 25 kDa and 250 kDa sodium hyaluronate solutions, two types of NPs were prepared by antisolvent precipitation in ethanol. The resulting NPs were dried in the presence of dipalmitoyl phosphatidylcholine, a natural synovial fluid component, then applied on an in vitro model of horse articular chondrocytes: no toxicity was observed and NPs prepared from 250 kDa HA promoted chondrocyte differentiation to a larger extent with respect to corresponding HA solutions, as evidenced by increased gene expression of chondrogenic markers (*Col2a1* and *Sox9*) and reduced expression of dedifferentiation markers (*Col1a1* and *Runx2*). These findings suggest that HA-based NPs are more effective at promoting the cellular internalization of the molecule and the differentiation of chondrocytes in vitro and could be a promising platform for drug delivery and cartilage repair.

## 1. Introduction

Cartilage is a tissue with reduced regeneration potential. Aging and pathological conditions, such as osteoarthritis (OA), require treatments to prevent and restore damages deriving from degeneration of cartilage and to provide palliation of pain. Apart from surgical and pharmacological treatments, cartilage can benefit from the administration of chondroprotective agents that help stimulate the synthesis of collagen, proteoglycans, and hyaluronic acid (HA); inhibit cartilage degradation; and prevent fibrin formation in the subchondral and synovial vasculature.

Among endogenous chondroprotective agents, HA is one of the most used in clinical practice. HA is a heteropolysaccharide composed of D-glucuronic acid and N-acetylglucosamine, linked by β(1–4) and β(1–3) glycosidic bonds, that, in joints, is mainly synthesized by type B synoviocytes, fibroblasts, and chondrocytes. It is one of the main components of synovial fluid and, together with the glycoprotein lubricin and with phospholipids like dipalmitoyl phosphatidylcholine (DPPC), is responsible for viscoelastic and lubricating properties of the fluid, including shock absorption. The biological activity of HA goes beyond lubrication and depends not only on its chemical structure but also on its molecular weight (MW) and concentration in synovial fluid [1]. The definition of HA as high, medium, or low molecular weight can be misleading and may vary by the context [2]. Here, we refer to HA above 1000 kDa as high molecular weight (HMW) and HA below 1000 kDa as low molecular weight (LMW). The biological effects of HA in the body are reported to be cell type- and context-specific [3], likely due to a differential expression of HA receptors. HAs of different molecular weights exert different effects on cell signalling and inflammation, both on synovial cells and on macrophages [4,5]. Regarding the cartilage, the literature on the biological effect of HA is conflicting, and the exact mechanisms of its chondroprotective effects are not fully clarified. For example, HA with a MW greater than 500 kDa, but not above 4000 kDa [6], is reported to promote endogenous production of high-MW HA, to interact with pain receptors, to inhibit the synthesis of pro-inflammatory mediators in joints, and to counteract mitochondrial DNA damage due to oxidative stress in human chondrocytes [7]. On the other hand, the reduction in MW from a starting weight of about 5–10 × 10^3^ kDa (typical of a healthy joint) to 500–3000 kDa is one of the symptoms of an arthritic condition, due to the effect of proinflammatory cytokines that induce its fragmentation [8,9]. In arthritic cartilage and in in vitro models of cartilage inflammation, LMW HA (20–122 kDa) is generally reported to stimulate pro-inflammatory cellular responses and to trigger a proinflammatory loop, promoting the progressive degeneration of cartilage; in any case, few reports describe its signalling effect after its addition in a non-inflammatory environment [10], and differences can occur even within the LMW family, as reported, for example, when considering HA oligomers [11]. The lubricating ability in synovial fluids after fragmentation is drastically reduced, but no consensus exists on the signalling effect of HA fragments on articular cartilage in physiological conditions. In fact, independently of its original size, after binding to the CD44 receptor, HA is cut by HYAL 2 into short chains of up to 20 kDa (approximately 50 disaccharide residues) to allow its internalization via receptor-mediated endocytosis [12,13,14], and further intracellular cleavage by HYAL1. Size reduction is also useful to improve the diffusion of the molecule into cartilage tissue, as demonstrated by Brackin et al. [15]. In this paper, methacrylated LMW HA, ranging between 20 and 100 kDa, showed a trend of greater material integration with lower molecular weight, with a superior chondroprotective activity by 20 kDa HA. Therefore, we hypothesized that LMW HA could exert a metabolic effect that we considered worth investigating since, to the best of our knowledge, no further literature is available describing the effect of LMW HA signalling on the expression of cartilage differentiation markers. Indeed, a critical problem in the practices of tissue regeneration of cartilage tissue lies in the fact that, after isolating chondrocytes for their in vitro expansion, dedifferentiation occurs, with a progressive increase in the expression of fibroblastic markers at every passage in culture.

We chose to explore two sizes of HA, the first the one (about 25 kDa) corresponding to the size of fragments produced by hyaluronidase digestion, and the second one, which is ten times larger (about 250 kDa), to have a significantly different size distribution, still within the low-molecular-weight range.

Moreover, since the signalling in response to HA depends not only on its molecular weight, but also on its concentration and its persistence in the site of application, we were interested in comparing any differences occurring by applying HA in solution or in the form of nanoparticles. The administration of HA as nanoparticles could provide more efficient delivery of the substance, extending its residence time and potentially constituting the basis for a targeted drug delivery system, by exploiting the binding of HA via CD44 to chondrocytes, that is expressed to a larger extent by subpopulations with high chondrogenic capacity [16,17,18]. Ideal particles for administration to the synovial membrane should have a size range between 5 and 300 nm so that they can pass the synovia and reach the cartilage. Ex vivo studies suggest that the cut-off to penetrate healthy cartilage is 55–140 nm [19], while osteoarthritic cartilage, due to corrosion, could be reached by NPs up to 300 nm [20]; apart from the size, the retention of nanomaterials is influenced by the pathological context as well [19]. If effective, HA-based nanoparticles could also constitute a drug delivery system with penetration potential to deliver drugs in deep ECM for maximum efficacy [21], overcoming one of the main problems associated with the intra-articular administration of HA in solution, namely, its quick degradation and high clearance in the joint cavity, rapidly reducing the duration of benefits deriving from the injection.

In this paper, we describe a mild method for the preparation of nanoparticles starting from HA of two low molecular weights, and their drying in association with DPPC, an amphiphilic lipid that is naturally present in synovial fluid and is reported to act in synergy in the lubrication of joints [22,23], to obtain a free-flowing and stable powder. The formulation as a dry powder, to be reconstituted right before injection, is expected to prolong both the chemical and microbiological stability of hyaluronic acid formulations. We report preliminary data on the effect of reconstituted powders applied in vitro to isolated horse articular chondrocytes by comparing them with the corresponding HA solutions, including the effect on cell morphology and expression of typical differentiation markers of cartilage.

## 2. Results

### 2.1. Characterization of NPs by DLS

Nanoparticles were prepared by antisolvent precipitation in ethanol by adapting a procedure previously described [24,25]. Different batches were prepared, to verify the reproducibility of the method (n = 7). The size of nanoparticles prepared starting from 15–30 kDa (referred to as 25NPs) or 100–300 kDa HA (referred to as 250NPs) was measured by DLS right after preparation and monitored in the following 10 days. The starting average particle size of freshly prepared 25NPs was 360 ± 31 nm, while for 250NPs, the starting size was 1015 ± 136 nm. The size of nanoparticles, stored at room temperature, was monitored for 10 days: both preparations showed a variation in size within 4 days. For 25NPs, this resulted in a slight reduction to 296 nm, while for 250NPs a significant reduction was observed, reaching a minimum of 253 nm. This time-dependent variation could be attributed to a conformational transition of HA molecules during the prolonged contact with ethanol. As also shown by NMR spectroscopy studies, in water, HA assumes a conformation that is strictly dependent on hydrogen bonding and results in a 2-fold helical symmetry, also exposing areas of hydrophobicity. The method of preparation of nanoparticles here described leads to a sudden desolvation of the polymeric chains, which leads to a rearrangement in which hydrophilic groups move inwards to minimize the contact with ethanol [26], while hydrophobic groups are exposed on the surface, thus enabling the interaction with lipophilic molecules as DPPC [27,28].

DPPC was added to the suspension in a 1:10 weight ratio to HA: this value is close to the physiological ratio of phospholipids and HA in normal synovial fluid (0.1–0.2 mg/mL for phospholipids and 1–4 mg/mL for HA [29]) and is reported to be the proper ratio for effective lubrication [30]. DPPC is an effective boundary lubricant of articulating surfaces and can act in synergy with HA to reduce friction in cartilage [23,30]. In this formulation, DPPC was intended to act not only as a potential ingredient with an ancillary role but also as a technological excipient to prevent the aggregation of NPs during the spray-drying step [31]. Before drying, the size of NPs was checked by DLS to verify if the addition of DPPC resulted in any significant change.

As can be seen in Table 1, no significant size variation was observed after the addition of DPPC to the nanosuspension. The ζ-potential of particles was measured as well, evidencing a difference between the two preparations: for both formulations, the sign was negative, but for 25NPs the modulus value was higher, suggestive of greater stability with respect to 250NPs. No variations in ζ-potential were observed after the addition of DPPC.

### 2.2. Drying of Nanosuspensions and Evaluation of Resulting Powder

Spray-drying of nanosuspensions was performed in the conditions described in the methods to remove ethanol from the preparation and allow its application to cell cultures. The yield of the process was 37 ± 3% for 25NPs and 44 ± 1% for 250NPs. The average size distribution (D_V50_) of resulting nano-aggregates, as assessed by laser diffraction analysis, was 1.394 μm (span 6.473) for 25NPs and 1.743 μm (span 2.545) for 250NPs.

### 2.3. Redispersion of Powders in Different Physiological Media

The behaviour of nanoparticles after reconstitution in physiological media can be significantly affected by ions and complex molecules, such as those present in cell culture media and, eventually, in synovial fluid. To this purpose the resulting powders were redispersed in PBS, DMEM, and DMEM + 10% FBS and, after sonication, the size of the resulting NPs was checked by DLS. Table 2 reports D_V50_ and standard deviation of NPs, showing that they recovered the size of the parent nanosuspension in physiological media to a different extent. In particular, 25NPs recovered their original size, despite a general increase in the width of the distribution. On the other hand, 250NPs behaved differently, depending on the redispersion medium: in PBS, the average size was higher but not significantly different from the original size, while in plain DMEM and in DMEM + 10% FBS different populations of particles were present. The first population, most abundant by number, had an average lower size (around 130 nm) while the second one, in lower percentage by number of nanoparticles, had a size largely above 500 nm. This can be ascribed to the different ζ-potential of the two formulations, 250NPs being more prone to aggregation because of their value closer to neutrality. Ionic strength is one of the main parameters affecting ζ-potential, but this is quite similar between PBS and culture media (152 mM and 169 mM, respectively), not justifying such difference. Another explanation could derive from the different structure and concentration of NPs after preparation: indeed, being comparable in size, the number of molecules per particle and polymer entanglement are different, and this can affect their drying kinetics. Likely, after redispersion of 250NPs in DMEM, the heads of some polymeric chains are exposed on particle surface making them available for interactions, leading to particle aggregation. Graphs showing the size distribution of particles are reported in the Supplementary Material (Figure S1).

### 2.4. Effects of NPs on Horse Chondrocytes

#### 2.4.1. Viability

**Figure 1:**
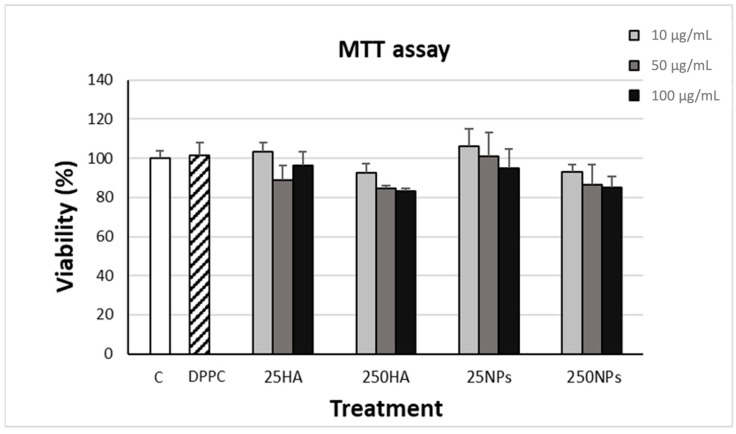
MTT assay for evaluating the viability of horse chondrocytes cultured in adhesion for 2 weeks with different concentrations (10, 50, and 100 µg/mL) of 25HA, 250HA, 25NPs, or 250NPs compared to non-treated cells (C) and DPPC solution (10 µg/mL). (Values are mean ± SD, n = 4/6).

MTT assay (Figure 1) was employed to evaluate chondrocyte viability for 2 weeks in the presence of the two molecular weights of HA in solution (25HA or 250HA) or formulated as NPs (25NPs and 250NPs). MTT did not show any significant difference at all the tested concentrations of HA and NPs as well as in the presence of DPPC at the highest concentration used in NPs (10 µg/mL), suggesting that no toxicity could be attributed to the presence of HA or DPPC as well as to the formulation process.

#### 2.4.2. Gene Expression Analysis of Cartilage Markers

**Figure 2:**
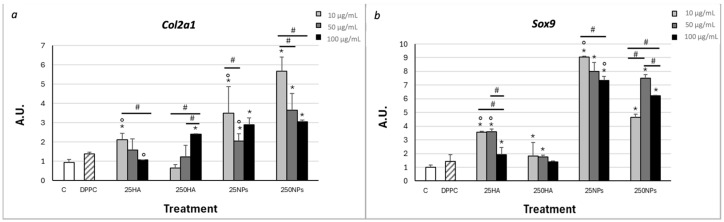
Gene expression of *Col2a1* (**a**) and *Sox9* (**b**) in horse chondrocytes cultured in adhesion for 2 weeks with different concentrations (10, 50, and 100 µg/mL) of 25HA, 250HA, 25NPs, or 250NPs compared to non-treated cells (C) and cells treated with DPPC in solution (10 µg/mL). * Statistically significant differences between each sample and the control. # Statistically significant difference between the different concentrations within each HA or NP treatment group. ° Statistically significant difference between the two different HA types or NP types within each concentration group. Values are mean ± SD, n = 4/6 (*p* < 0.05).

Gene expression of *Col2a1* (Figure 2a) was comparable to control in the presence of both types of HA in solution, with a slight increase of 10 µg/mL for 25HA and 100 µg/mL for 250HA. *Col2a1* gene expression was significantly higher in the presence of both types of NPs compared to control at all concentrations tested.

Gene expression of transcription factor *Sox9* (Figure 2b) was significantly increased with both HA solutions and NPs, but the increase was much higher when NPs were applied, independently of the molecular weight of HA, at all three concentrations of NPs.

The administration of DPPC in solution at the highest concentration present in NPs did not determine a significant difference in gene expression, thus allowing us to exclude any interference in gene expression ascribable to the presence of this excipient. These data suggest that the formulation of HA as nanoparticles improves the ability of the molecule to exert its signalling function, probably by increasing locally its concentration on the membrane to bind CD44 [32]. It is not clear why this effect is not related to the applied concentration of nanoparticles, but this could be reasonably ascribed to a saturation of the receptors blocking its internalization by endocytosis, as previously reported [14].

**Figure 3:**
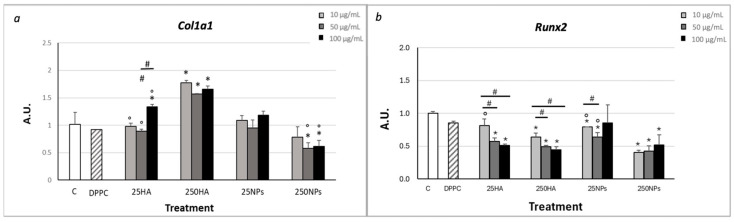
Gene expression of *Col1a1* (**a**) and *Runx2* (**b**) in horse chondrocytes cultured in adhesion for 2 weeks with different concentrations (10, 50, and 100 µg/mL) of 25HA, 250HA, 25NPs, or 250NPs compared to non-treated cells (C) and cells treated with DPPC in solution (10 µg/mL). * Statistically significant differences between each sample and the control. # Statistically significant difference between the different concentrations within each HA or NPs treatment group. ° Statistically significant difference between the two different HA types or NP types within each concentration group. Values are mean ± SD, n = 4/6 (*p* < 0.05).

Gene expression of Col1a1 (Figure 3a) did not show statistically significant differences between control and 10 and 50 µg/mL of 25HA, but it was slightly increased only with 100 µg/mL; differently, the expression was significantly increased in the presence of all concentrations of 250HA. Non-statistically significant differences were observed between control and 25NPs; more interestingly, the addition of 250NPs reduced gene expression of dedifferentiation marker *Col1a1* at all concentrations used.

Gene expression of *Runx2* (Figure 3b) was significantly reduced in all the tested conditions compared to control, particularly with 250NPs.

#### 2.4.3. Immunofluorescent Staining

**Figure 4:**
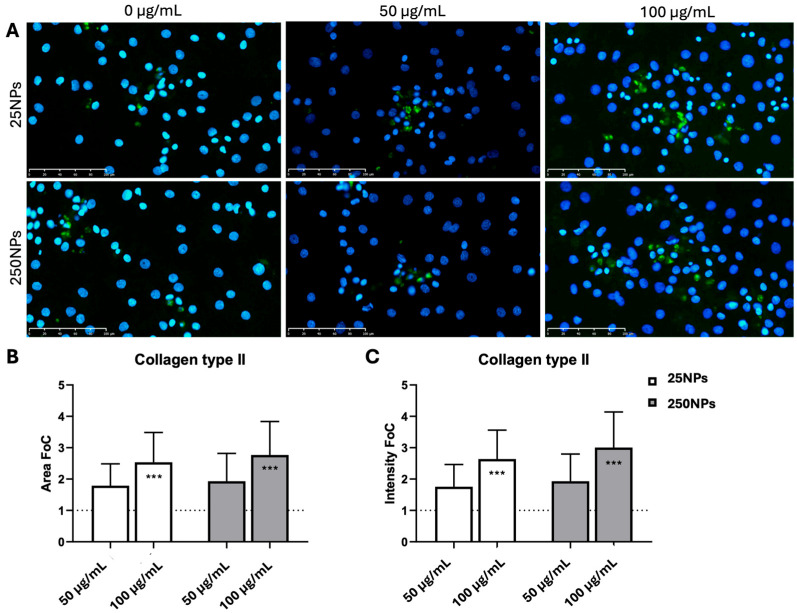
(**A**) Immunofluorescence staining for collagen type II on primary chondrocytes after 2 weeks of culture in adhesion with different concentrations (0, 50, and 100 µg/mL) of 25HA NPs or 250HA NPs. Collagen type II—FITC (green); Nuclei—DAPI (blue). Scale bar = 100 μm. Histograms of collagen type II quantification in the various conditions: (**B**) area and (**C**) intensity of IF marker detection express as fold of change; 25NPs are in blank, 250NPs are in grey (*** *p* < 0.001).

Collagen type II was selected as a specific chondrogenic marker of extracellular matrix production. The cell positivity to collagen type II was preferentially located in the extracellular space next to cell clusters rather than in single isolated cells (Figure 4A). The quantification of the area and the intensity of the marker collagen type II after 2 weeks of culture revealed that with both types of NPs at the concentration of 100 µg/mL, a significantly higher level of protein was present as compared with the control (Figure 4B,C). The concentration of 50 µg/mL of both types of NPs determined an increase in the chondrogenic marker too, but without a statistically significant difference with respect to the control (Figure 4B,C).

#### 2.4.4. Scanning Electron Microscopy

**Figure 5:**
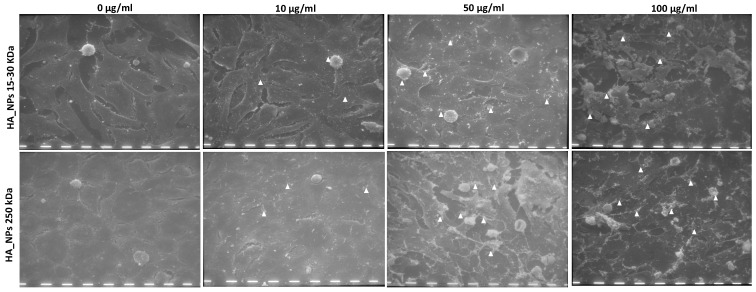
Scanning electron microscopy (SEM) of primary chondrocytes after 2 weeks of culture in adhesion with different concentrations (0, 10, 50, and 100 µg/mL) of 25NPs or 250NPs. The arrowheads indicate the secreted extracellular matrix. Scale bar = 10 μm.

The considerations made from immunofluorescent staining were also confirmed by morphological observations of cells with SEM. The pictures in Figure 5 evidenced the effect of NPs on cell morphology and matrix production; in fact, for both 25NPs and 250NPs, the increasing concentration of NPs was positively associated with increased production of extracellular matrix and with the appearance of a roundish cell morphology associated with chondrocytes in differentiated state. The extracellular matrix deposition resulted in the formation of separate short filaments for both types of NPs at the concentration of 10 µg/mL, which became a filamentous network at the concentrations of 50 and 100 µg/mL of NPs. In the presence of 250NPs, cells appeared more roundish and surrounded by a higher amount of extracellular matrix.

These data suggest that both types of NPs prepared from LMW hyaluronate, and in particular 250NPs, can exert a metabolic effect on chondrocytes, promoting the expression of genes that positively contribute to the preservation of a differentiated phenotype and the production of extracellular matrix proteins.

## 3. Discussion

Significant research efforts are devoted to producing engineered cartilage as a cell-based approach for articular repair because articular cartilage has a poor intrinsic healing capacity after joint injury. For tissue engineering applications, autologous chondrocytes obtained from the healthy zone are cultured and expanded in vitro, but in this condition, it is difficult to maintain the chondrogenic phenotype to produce ECM with adequate mechanical properties [33,34]. Numerous microenvironmental factors (pH, O_2_, osmolarity, and growth factors) have been studied for improving the quality of the matrix produced in vitro [35]. HA is also widely used in vivo by intra-articular injection and in vitro, in association with other materials, to create biomaterials to maintain the chondrocyte phenotype [36].

In this paper, we propose a mild formulation approach for the preparation of HA-based nanoparticles by antisolvent precipitation in ethanol. The procedure has the advantage that no structural modifications occur on HA, that could potentially modify the chemical nature of the molecule and disrupt its ability to bind to its receptor [37]. The resulting nanoparticles have an adequate size to be internalized by articular chondrocytes [19,20]. Drying was necessary to remove ethanol from the preparation in view of their application in vitro. No bulking agents were introduced in the formulation: the only excipient was DPPC, which was added in a 1:10 weight ratio with respect to HA, similar to the physiological ratio of phospholipids and HA in normal synovial fluid. Apart from its physiological role, DPPC was expected to prevent powder aggregation during drying and help in its redispersion in different media.

Powders of 25NPs were efficiently redispersed in all media tested, recovering their original size, while 250NPs showed a different behaviour according to the nature of the dispersing medium. In particular, with DMEM, independent of the presence of serum, two populations appeared: one slightly smaller than the original one and a larger one, above 500 nm in diameter. Likely, some components of DMEM interact preferentially with hyaluronate molecules, hydrating nanoparticles faster than other media. It is interesting to notice that this only happened with 250NPs, suggesting that the structure of NPs or the folding of hyaluronate during precipitation changes according to the molecular weight of the polymer, probably due to a different ζ-potential and drying kinetic.

For preliminary in vitro tests with HA-based NPs, we used primary proliferating chondrocytes in adhesion cultures which is an optimal model to study the micro-environmental signals influencing chondrocyte differentiation. In fact, it is well known that when chondrocytes are grown in primary adhesion culture under standard physiological conditions (foetal calf serum and 20% O_2_), a de-differentiation process starts with a quick and drastic decrease of *Col2a1* and *Sox9* gene expressions and an increase in de-differentiation markers such as *Col1a1* and *Runx2* [38], and this de-differentiated phenotype is also typical of chondrocytes during OA [39].

At first, the MTT assay was set up to exclude any potential cytotoxicity deriving from the addition of DPPC and HA in solution or in the form of NPs at three increasing concentrations. The obtained results indicated a high biocompatibility of all considered conditions, showing no reduction of viability with respect to control.

*Col2a1* and *Sox9* were selected as markers of chondrogenic differentiation. PCR analysis revealed that when both types of NPs were added to the culture medium, an increase in gene expression of *Col2a1* and *Sox9* occurred in comparison to control; this increase was much higher than that observed when HA was applied in solution, suggesting that the formulation as nanoparticles improved HA’s ability to bind to CD44 and probably the internalization of the polymer.

Conversely, *Col1* and *Runx2* were selected as markers of chondrogenic de-differentiation. Interestingly, *Runx2* gene expression showed a significant decrease with 25 kDa HA and 250NPs, and the values of this gene expression were significantly lower in the presence of 250 kDa HA and particularly with 250NPs. For the HA condition, the behaviour of *Col1a1* is different and does not appear to be in parallel with *Runx2*: indeed, with this treatment, the expression of *Col1a1* was increased, while in the presence of NPs at high MW, *Col1a1* expression was lower in comparison to the control.

These data suggest that low-molecular-weight HA, in particular in the form of nanoparticles, can have a positive effect on the expression of differentiation markers, which could be explained by a higher local concentration of the polymer on the cell surface, or by a peculiar folding of the molecule after desolvation with ethanol that exposes specific groups of the polymer, favouring its interactions with CD44 clusters and its consequent internalization. It is not excluded that DPPC could exert a role in helping the internalization of HA, but this should be further investigated. Moreover, it was excluded that the effects on gene expression could be ascribed to the presence of the lipid, as demonstrated by the control experiments in which DPPC only was added to the culture medium, without any effect. Curiously, no clear trends in the modulation of gene expression could be identified in relation to different concentrations of hyaluronate applied, independent of its use as a solution or as nanoparticles. At the protein expression level, the higher concentration of 250NPs (100 µg/mL) led to a higher number of collagen type II-producing cells with respect to the control and to other concentrations, and this also corresponded to a greater matrix deposition and the acquisition of a more roundish morphology, typical of differentiated chondrocytes.

In this preliminary cell model, NPs, particularly if prepared with 250 kDa HA, proved to represent an effective formulative approach for maintaining chondrogenic differentiation and chondrocyte metabolism.

## 4. Materials and Methods

### 4.1. Materials

Sodium hyaluronate having molecular weights ranging between 15 and 30 kDa and 130 and 300 kDa was obtained from Contipro (Praha, Czech Republic). Ultrapure water was obtained by inverse osmosis using an Arium Comfort^®^ system by Sartorius (Gottingen, Germany). 1,2-Dipalmitoyl-sn-glycero-3-phosphocholine (DPPC) 16:0/16:0 was from Lipoid GmbH (Ludwigshafen, Germany).

### 4.2. Production of Sodium Hyaluronate Nanoparticles

The whole procedure was performed with sterile glassware under a vertical laminar flow hood. Sodium hyaluronate nanoparticles (NPs) were obtained by anti-solvent precipitation in ethanol of a hyaluronate solution. For the preparation of nanoparticles, 200 mg of 15–30 kDa or 130–300 kDa sodium hyaluronate were dissolved in 40 mL or 20 mL, respectively, of ultrapure sterile water under stirring. After complete dissolution, 160 mL or 120 mL of ethanol, respectively, were suddenly added to the solution, to induce hyaluronate precipitation. The resulting nanosuspensions were coded as 25NPs or 250NPs.

### 4.3. Characterization of NPs by Dynamic Light Scattering (DLS) and z-Potential

The hydrodynamic diameter and polydispersity index of NPs were determined by dynamic light scattering, using a Nanozetasizer ZS (Malvern Panalitycal Ltd., Malvern, UK), which operates with a laser source of He–Ne (λ = 633 nm and P = 5 mW). The scattered light was measured at a fixed angle (*θ*) of 173° and a temperature of 25 °C. The size of nanoparticles was measured in triplicate (n = 3) after 1:4 dilution in ethanol 80% *v*/*v*. The same instrument was used to determine the z-potential of nanoparticles after preparation.

### 4.4. Drying of Nanoparticles

The nanosuspensions were dried using a spray drier Büchi B-290 (Büchi AG, Flawil, Switzerland) equipped with an Inert Loop B-295 to operate in closed-loop mode. A 0.7 mm nozzle was mounted, inlet temperature was set at 90 °C, nebulization of nitrogen was 700 L/h, aspiration flow rate 28 m^3^/h, and pump feed rate was 3 mL/min. Before drying, an aliquot of DPPC solution (10 mg/mL) in ethanol was added to each nanosuspension, in a 1:10 weight ratio of DPPC to sodium hyaluronate. The resulting powder was then collected under a laminar flow hood to prevent microbial contamination.

### 4.5. Evaluation of Particle Size Distribution of the Powder

The particle size distribution of the resulting powder was determined with a diffractometer Spraytec (STP5321, Malvern Panalitycal Ltd., Malvern, UK) equipped with a 300 mm focal lens and a wet cell. Ten milligrams of each sample was dispersed in a solution of cyclohexane (VWR International, Milan, Italy) with 0.1% *v/v* Span 85^®^ (Merck KGaA, Darmstadt, Germany) and sonicated for 2 min right before measurement with a threshold obscuration of at least 7%.

### 4.6. Redispersion of the Powder

The resulting powder was resuspended in different buffers to evaluate the ability of NPs to recover their original size. First, 1 mg of powder was redispersed in 4 mL of phosphate-buffered saline (PBS), or Dulbecco’s modified Eagle medium (DMEM), at normal or high glucose. To help redispersion of the powder, sonication was performed with a Branson 2510 ultrasonic bath.

### 4.7. Cell Cultures

#### 4.7.1. Establishment of Primary Culture of Horse Chondrocytes

Fetlock joints of horses slaughtered for human consumption were immediately delivered to the laboratory and then used to collect the cartilage. Before cartilage harvesting, joints were carefully examined and those with macroscopic lesions related to overt osteoarthritis or with evidence of synovitis were excluded from the study. Chondrocytes were isolated from the articular cartilage and cultured following a protocol previously described [40,41]. Briefly, cartilage was finely diced under sterile conditions and then washed several times in sterile phosphate-buffered saline (PBS). After pre-incubation in 0.1% pronase (Sigma-Aldrich, St. Louis, MO, USA) solution for 1 h at 37 °C, the tissue was treated with 0.2% collagenase type IA (Sigma-Aldrich, St. Louis, MO, USA) in DMEM (4.5 g/L glucose; 25 mM Hepes) for 2 h at 37 °C. The digested material was filtered through 100 µm and 20 µm nylon filters, and the cellular suspension was centrifuged at 1800 rpm for 10 min. The supernatant was discarded, and the pellet was washed several times with DMEM containing 10% foetal bovine serum (FBS), 100 U/mL penicillin, and 0.1 mg/mL streptomycin (complete medium). The number of chondrocytes was determined using a hemocytometer (Marienfield, Lauda-Königshofen, Germany), and the cell viability (never less than 95%) was assessed by trypan blue (0.1%) exclusion.

Isolated chondrocytes were seeded in adhesion on plastic in Petri dishes at a density of 5 × 10^4^ cells/cm^2^ in a complete medium with 50 µg/mL ascorbic acid.

All cultures were incubated at 37 °C in a humidified atmosphere of 5% CO_2_ for the time specified for every single experiment and the medium was exchanged biweekly.

Dried NPs were accurately weighed and resuspended in a serum-free medium and sonicated for 5 min at 37 °C before applying them to cell cultures at a concentration of 10, 50, or 100 μg/mL.

#### 4.7.2. MTT Assay

Cell viability was evaluated by measuring the mitochondrial dehydrogenase activity using a modified MTT (3-(4,5-dimethyl-2-thiazolyl)-2,5-diphenyl-2H-tetrazolium bromide) reduction assay (Roche Applied Science, Penzberg, Germany). Briefly, two weeks after seeding on dishes, the cells were incubated with 250 µL of complete DMEM and 25 µL of MTT solution (Sigma-Aldrich, St. Louis, MO, USA) in 5 mg/mL PBS for 4 h at 37 °C. Then, 250 µL of 10% SDS in 0.01 M HCl was added and incubated overnight at 37 °C. The resulting solution was moved onto fresh plates, and the absorbance was measured using the multimode plate reader VICTOR Nivo (PerkinElmer, Waltham, MA, USA) at a wavelength of 570 nm. The absorbance read in wells without cells with medium and MTT was used as a control sample (blank).

### 4.8. Gene Expression Analysis of Differentiation Markers

#### 4.8.1. Total RNA Extraction and cDNA Synthesis

The total RNA of each sample was extracted using TRIreagent (Thermo Fisher Scientific, Waltham, MA, USA), according to the manufacturer’s instructions. Purity and concentration were assessed by UV spectrophotometry at 260/280 nm and 260 nm, respectively (BioSpectrometer, Eppendorf, Hamburg, Germany). RNA integrity and quality were assessed by using an Agilent Bioanalyzer 2100 and RNA 6000 Labchip kit (Agilent Technologies, Santa Clara, CA, USA).

#### 4.8.2. Reverse Transcription (RT)

All RNA samples were DNAse-treated (Sigma-Aldrich, St. Louis, MO, USA) before cDNA synthesis. Total RNA (1 μg/20 μL) was reverse-transcribed using a High-capacity cDNA Reverse Transcription kit (Applied Biosystems-Life Technologies, Carlsbad, CA, USA). The Reverse Transcription (RT) was performed according to the manufacturer’s instructions, under the following thermal conditions performed by using a StepOne thermocycler (Applied Biosystems, StepOne): 10 min at 25 °C, 120 min at 37 °C, followed by 5 min at 85 °C. All cDNA samples were stored at −20 °C until PCR was performed. The cDNA obtained was used as a template for a subsequent polymerase chain reaction.

#### 4.8.3. Quantification of mRNA by Real-Time PCR (qPCR)

cDNA concentration was assessed by UV-spectrophotometry (BioSpectrometer, Eppendorf, Hamburg, Germany), and 5 ng of each sample was used as a template for real-time quantitative PCR (qPCR) performed using a StepOne thermocycler (Applied Biosystems, StepOne software v. 2.1). The cDNA (5 ng/20 μL) was amplified in triplicate with Power Up SYBR Green Master Mix (Applied Biosystems-Life Technologies, Carlsbad, CA, USA) along with specific sets of primers at 300 or 500 nM. The primers were designed based on published gene sequences or by using the Primer Express^®^ software package (v. 3.0, Applied Biosystems) to create oligonucleotides with similar melting temperatures and minimal self-complementary and purchased from Eurofins Genomics (Ebersberg, Germany). Details of each primer set for the detection of gene expression are reported in Table 3.

The reference gene Gapdh was selected as endogenous control according to minimal intra-/inter-assay variation. Samples were kept at 95 °C for 20 s (hold step) to allow DNA-polymerase activation and then subjected to 40 cycles consisting of a denaturation step at 95 °C for 3 s followed by an annealing/extension step at 60 °C for 30 s. Fluorescence due to SYBR Green I incorporation was acquired at the end of the extension step. A no-RT control and a no-template control (NTC) were included in each experiment. A melting curve analysis for specific amplification control was performed (from 60 °C to 95 °C) at the end of the amplification cycles. NTC controls were assumed as negative and reliable if the quantification cycle (Cq) was ≥35. Data were analysed according to the 2^−ΔΔCt^ method [42,43] in which expression levels of each gene were normalized to the Gapdh cDNA amount and expressed as relative quantities.

### 4.9. Immunofluorescent Staining

Immunofluorescent (IF) reactions were performed on Chamber slide seeding with a concentration of 0, 10, 50, or 100 mg/mL of NPs 15–30 kDa NPs or 250 kDa NPs. Briefly, after 2 weeks of culture, cells were fixed, permeabilised with 0.1% Triton (Sigma-Aldrich, St Louis, MO, USA), and rinsed in PBS (Sigma-Aldrich, St Louis, MO, USA) at room temperature. Sections were incubated overnight at 4 °C with primary antibody (Biorbyt, Cambridge, UK, Collagen II antibody: 2 µg/mL, Orb156420). The reaction was revealed by 1 h incubation at room temperature with specific secondary antibody (Alexa Fluor 488, Goat anti-Rabbit IgG (H + L), dilution 1:500, code 111-545-144, Jackson ImmunoResearch, Ely, UK). Finally, the nuclei were counterstained with DAPI (Thermo Fisher Scientific, Waltham, MA, USA). For negative control, the primary antibody was omitted, and cells were incubated in 10 mM phosphate buffer. Fluorescent Whole Slide Images were acquired using the NanoZoomer S60 slide scanner (Hamamatsu Photonics K.K., Hamamatsu City, Japan). Quantification of collagen type II deposition was performed on 6 ROIs (600 × 400 μm) through Qpath software (version 5.0).

### 4.10. Scanning Electron Microscopy

The scanning electron microscopy (SEM) of cells grown in the presence of nanoparticles was performed after 2 weeks of culture. At each selected time point, cells were fixed with 2.5% glutaraldehyde in 0.1 M sodium cacodylate buffer (pH 7.3) for 30 min at 4 °C. They were then dehydrated through a series of increasing grades of ethanol (from 50% *v/v* until absolute) and then critical point dried with liquid carbon dioxide (CPD 030 Baltec, Wallruf, Germany). Specimens were then sputter-coated with gold–palladium (Plano GmbH, Wetzlar, Germany) using an SCD 040 coating device (Balzer Union, Wallruf, Germany). The samples were observed using a Philips 501 SEM scanning electron microscope (Philips, Eindhoven, The Netherlands) at an accelerating voltage of 5 kV. Cells cultivated without nanoparticles were used as control samples (blank).

## Figures and Tables

**Table 1:** Evaluation of the size and ζ-potential of hyaluronate-based NPs with or without DPPC.

MW of HA (kDa)	Before DPPC		After DPPC Addition	
	Size (nm)	ζ-Potential (mV)	Size (nm)	ζ-Potential (mV)
25	326 ± 38	−21.6 ± 15.8	296 ± 21	−20 ± 14
250	268 ± 48	−11.5 ± 16.5	271 ± 15	−12.1 ± 11.8

**Table 2:** Size of NPs (average diameter ± standard deviation) after reconstitution of powders in different physiological media. When multiple populations were present, the most representative by intensity were reported. Polydispersity index is in brackets.

HA MW (kDa)	Before Drying (nm)	PBS (nm)	DMEM (nm)	DMEM + 10% FBS (nm)
25	228 ± 99 (0.171)	229 ± 209 (0.424)	225 ± 65 (0.323)	291 ± 113 (0.606)
250	271 ± 15 (0.388)	327 ± 57 (0.495)	138 ± 33 1081 ± 423 (0.725)	119 ± 46 543 ± 227 (0.645)

**Table 3:** Primer sequences used for real-time polymerase chain reaction: *Gapdh* (Glyceraldehyde 3-phosphate dehydrogenase); *Col1a1* (Collagen type I); *Col2a1* (Collagen type II); *Sox9* (Transcription factor SOX-9) [41].

Gene	Primer Sequences	Primer Concentration (nM)
*Gapdh*	Fwd: CAAGGCTGTGGGCAAGGT	300
	Rev: GGAAGGCCATGCCAGTGA	300
*Col1a1*	Fwd: AGAAGAAGACATCCCAGCAGTCA	500
	Rev: CAGGGCTCGGGTTTCCATA	500
*Col2a1*	Fwd: CTGGTGATGATGGTGAAG	300
	Rev: GTAACCTCTGTGACCTTTG	300
*Sox9*	Fwd: CAGGTGCTCAAGGGCTACGA	300
	Rev: GACGTGAGGCTTGTTCTTGCT	300

## Data Availability

Data are available upon request to the corresponding author.

## Supplementary Materials

The following supporting information can be downloaded at: https://www.mdpi.com/article/10.3390/ijms252312486/s1.
