# Supplementary material for: Effect of Low-Molecular-Weight Hyaluronate-Based Nanoparticles on the In Vitro Expression of Cartilage Markers
Source: Int J Mol Sci. 2024 Nov 21;25(23):12486. doi: 10.3390/ijms252312486 (PMC11641601; doi:10.3390/ijms252312486)

Figure S1: Particle size distribution analysis of NPs after reconstitution of powders in different physiological media

a) 25NPs Before drying

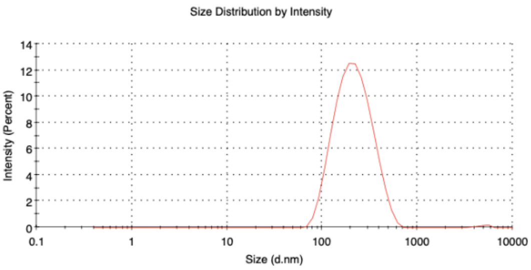

b) 25NPs redispersed in PBS

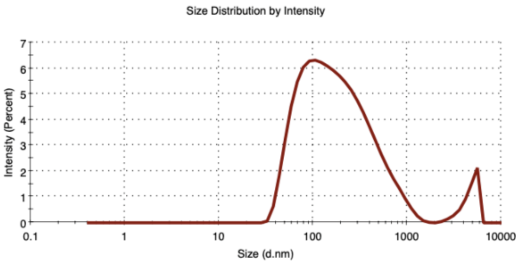

c) 25NPs redispersed in DMEM

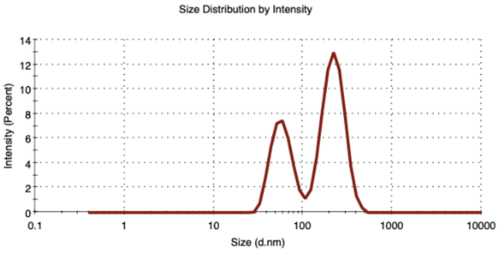

d) 25NPs redispersed in DMEM+ 10% FBS

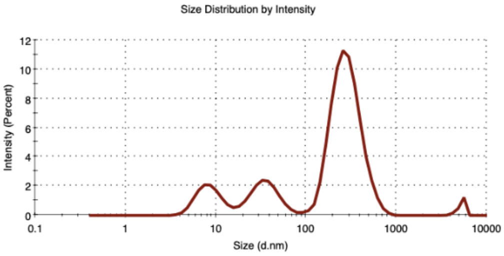

e) 250NPs Before drying

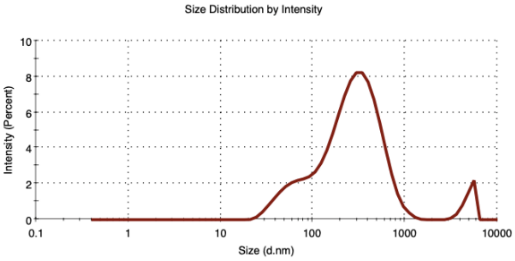

f) 250NPs redispersed in PBS

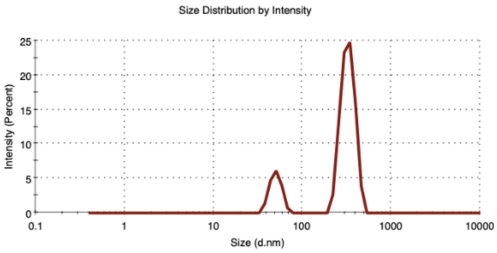

g) 250NPs redispersed in DMEM

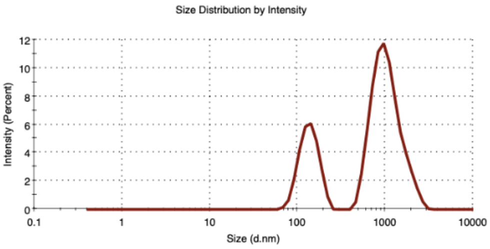

h) 250NPs redispersed in DMEM+ 10% FBS

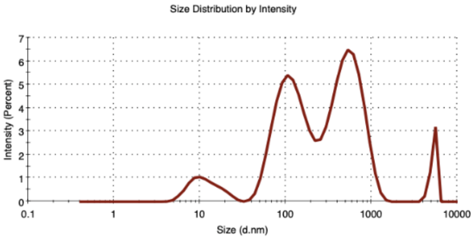

Supplement: Supplementary file 1 [file ijms-25-12486-s001.zip › ijms-3287880-supplementary.pdf]
